# FFAR1 activation attenuates histamine-induced myosin light chain phosphorylation and cortical tension development in human airway smooth muscle cells

**DOI:** 10.1186/s12931-020-01584-w

**Published:** 2020-11-30

**Authors:** Shengjie Xu, Anthony Schwab, Nikhil Karmacharya, Gaoyuan Cao, Joanna Woo, Nicholas Kim, Steven S. An, Reynold A. Panettieri Jr, Joseph A. Jude

**Affiliations:** 1 The Joint Graduate Program in Toxicology, Department of Pharmacology & Toxicology, Ernest Mario School of Pharmacy, Piscataway, USA; 2 Rutgers Institute for Translational Medicine & Science, New Brunswick, NJ 08901 USA; 3 Department of Pharmacology, Rutgers-Robert Wood Johnson Medical School, Rutgers, The State University of New Jersey, Piscataway, NJ 08854 USA; 4 Pharmacology & Toxicology, Ernest Mario School of Pharmacy, Rutgers, The State University of New Jersey, Rm: 4276, 89, French Street, New Brunswick, NJ 08901 USA

**Keywords:** Airway smooth muscle, Free fatty acid receptor 1, Airway hyperresponsiveness

## Abstract

**Background:**

Activation of free fatty acid receptors (FFAR1 and FFAR4) which are G protein-coupled receptors (GPCRs) with established (patho)physiological roles in a variety of obesity-related disorders, induce human airway smooth muscle (HASM) cell proliferation and shortening. We reported amplified agonist-induced cell shortening in HASM cells obtained from obese lung donors. We hypothesized that FFAR1 modulate excitation–contraction (EC) coupling in HASM cells and play a role in obesity-associated airway hyperresponsiveness.

**Methods:**

In HASM cells pre-treated (30 min) with FFAR1 agonists TAK875 and GW9508, we measured histamine-induced Ca^2+^ mobilization, myosin light chain (MLC) phosphorylation, and cortical tension development with magnetic twisting cytometry (MTC). Phosphorylation of MLC phosphatase and Akt also were determined in the presence of the FFAR1 agonists or vehicle. In addition, the effects of TAK875 on MLC phosphorylation were measured in HASM cells desensitized to β_2_AR agonists by overnight salmeterol treatment. The inhibitory effect of TAK875 on MLC phosphorylation was compared between HASM cells from age and sex-matched non-obese and obese human lung donors. The mean measurements were compared using One-Way ANOVA with Dunnett’s test for multiple group comparisons or *Student’s* t-test two-group comparison. For cortical tension measurements by magnetic twisted cytometry, mixed effect model using SAS V.9.2 was applied. Means were considered significant when p ≤ 0.05.

**Results:**

Unexpectedly, we found that TAK875, a synthetic FFAR1 agonist, attenuated histamine-induced MLC phosphorylation and cortical tension development in HASM cells. These physiological outcomes were unassociated with changes in histamine-evoked Ca^2+^ flux, protein kinase B (AKT) activation, or MLC phosphatase inhibition. Of note, TAK875-mediated inhibition of MLC phosphorylation was maintained in β_2_AR-desensitized HASM cells and across obese and non-obese donor-derived HASM cells.

**Conclusions:**

Taken together, our findings identified the FFAR1 agonist TAK875 as a novel bronchoprotective agent that warrants further investigation to treat difficult-to-control asthma and/or airway hyperreactivity in obesity.

## Introduction

Obesity contributes to asthma diathesis by enhancing airway hyperresponsiveness (AHR) and attenuating the response to standard asthma therapy [1–3]. Human airway smooth muscle (HASM) cells play pivotal roles in asthma through its contractile, immunomodulatory and remodeling functions [4, 5]. We previously reported that HASM cells from obese lung donors show amplified cell shortening in response to contractile agonists [6]. Contractile agonists elevate intracellular calcium ([Ca^2+^]_i_) levels, activate myosin light chain kinase (MLCK), and increase the phosphorylation of myosin light chain (MLC), promoting cross-bridge formation and ASM cell shortening [7]. In a parallel mechanism, myosin light chain phosphatase (MLCP) is inhibited through RhoA-activated Rho-associated kinase (ROCK), maintaining increased MLC phosphorylation (pMLC) levels in HASM cells [8, 9]. Additionally, RhoA can activate actin polymerization and focal adhesion protein paxillin phosphorylation and, thereby, reinforcing mechanotransduction through the cell surface integrin receptors and cortical tension development [10]. How obesity affects these surrogate measures (i.e. [Ca^2+^]_i_, pMLC, and paxillin phosphorylation) of excitation–contraction (E–C) coupling in HASM shortening is unclear. It is equally unclear the structure–function relationship between an altered E–C coupling and obesity-associated AHR.

Lipid mediators play pivotal roles in various aspects of asthma pathogenesis. Cysteinyl leukotrienes, generated from arachidonic acid metabolism, modulate AHR and leukotriene inhibitors are used as therapeutics in asthma patients [11, 12]. There is a renewed interest in other lipids and their mechanisms of action on airway structural cells [13]. Fatty acid receptors are expressed in a variety of tissues and modulate various cellular functions. Free fatty acid receptor 1 (FFAR1), also known as GPR40, is a de-orphaned G protein-coupled receptor (GPCR) expressed in a variety of tissues [14]. FFAR1, activated by long-chain fatty acid linoleic acid, is reportedly linked to Gα_q_ [14]. In guinea pig bronchial rings, activation of FFAR1 has been shown to potentiate acetylcholine-induced contraction [15].

Serum levels of free fatty acids are elevated in obesity and modulate systemic inflammation associated with the metabolic syndrome (metabolic inflammation) [16]. These fatty acids, in addition to acting as biomarkers of dyslipidemia in obesity, play (patho)physiological roles in several organ systems in obese subjects. Supporting the negative impact of elevated plasma free fatty acids, studies found that high-fat diet acutely increases airway inflammation and attenuates bronchodilator response in asthma patients [17]. However, it appears that not all free fatty acids negatively impact lung functions. A recent study found that FFAR4 (GPR120), a long chain free fatty acid receptor, mediates ASM relaxation [18]. Accordingly, we posit that free fatty acid receptors (FFAR1 and FFAR4) can modulate E–C coupling in HASM cells and play a role in obesity-associated AHR. Unexpectedly, our findings show that TAK875, a pharmacological agonist for FFAR1, but not GW9508 that targets both FFAR1 and FFAR4, attenuated histamine-evoked cortical tension development in HASM cells. Inhibition of tension was associated with decreases in MLC phosphorylation, but not calcium flux, phosphorylation of AKT or MYPT1. Of note, FFAR1 agonist-mediated inhibition of MLC phosphorylation was operational with carbachol stimulation, maintained in β_2_AR-desensitized HASM cells, and across obese and non-obese donor-derived HASM cells. These findings warrant further investigation on FFAR1 agonists as novel bronchoprotective agents.

## Material and methods

### Reagents

HAM’s F-12 medium, PBS, FBS, 0.05% Trypsin and EDTA, and PAGE/western blotting were purchased from Life Technologies (Carlsbad, CA). Antibodies for pMLC (pS^18^/T^19^-MLC), total MLC, p^S507^MYPT1, total MYPT1, pAKT, total AKT and tubulin were purchased from Cell Signaling Technology (Danvers, MA). Fluo-8 calcium flux assay kit was purchased from Abcam (Cambridge, MA). GW9508 and TAK875 were purchased from Cayman Chemical Company (Ann Arbor, MI). Cyclic AMP-Screen assay kit was purchased from Applied Biosystems (Bedford, MA).

### Culture of HASM cells

Primary HASM cells were harvested, characterized and grown in culture as described by us in detail previously [19]. For all experiments, cells were used within the first 4 passages to ensure proper smooth muscle phenotype. HASM cells were serum-deprived 48 h prior to experimental exposures.

### Exposure to testing compounds

HASM cells were exposed to compounds in F-12 culture medium without serum. GW9508 and TAK875 were dissolved in DMSO. All subsequent dilutions were made in serum-free F-12 medium. GW9508 or TAK875 were initially used at 0.1–10 μM concentrations for 10 and 30 min to detect the effect on pMLC level. In subsequent experiments, both compounds were used at 10 μM for 30 min. The DMSO concentration in the vehicle control was 0.05–0.1%. To determine MLC, MYPT1 or AKT phosphorylation, HASM cells were exposed to 25 μM carbachol (CCh) or 2.5 μM histamine for 10 min. Cell lysates were collected in 0.6 M HClO_3_ to precipitate proteins.

### Magnetic twisting cytometry

We used magnetic twisting cytometry (MTC) to measure dynamic changes in the cytoskeletal stiffness as a surrogate for agonist-induced force generation at the single-cell level. An RGD-coated ferrimagnetic microbead functionalized to the cytoskeleton through cell surface integrin receptors was magnetized, twisted by an external magnetic field that varied sinusoidally in time, and forced bead motions were detected as previously described [20]. Cell stiffness is expressed as Pascal per nm. We applied mixed effect model using SAS V.9.2. and report estimated mean ± SEM.

### Measurement of [Ca^2+^]_i_ in HASM cells

Agonist-induced [Ca^2+^]_i_ in HASM cells was determined as previously described with some modifications [21]. Briefly, HASM cells grown to confluence in a 48-well plate were loaded with fluo-8 Ca^2+^-binding dye. Carbachol (25 μM) or histamine (2.5 μM) were used to elicit Ca^2+^ response in HASM cells. Fluorescence intensity was monitored for up to 100 s following agonist injection. Area under the curve (AUC) of the time-dependent fluorescence (relative fluorescence units-RFU) was calculated from the response curve.

### Measurement of cyclic adenosine monophosphate (cAMP) in HASM cells

HASM cells were seeded and grown in a 24‐well plate until about 80% confluent before serum-withdrawal. Cells were stimulated, lysed and analyzed by cAMP screen ELISA system by Applied Biosystems (Bedford, MA) following manufacturer’s instructions.

### Data analysis

HASM cells from at least 5 donors (Additional file 1: Table S1) were used in the experiments. When applicable, the experimental readouts were first normalized to vehicle control in each donor to obtain the fold change. The fold changes from individual donors were used to obtain group mean graphs. The data are expressed as mean ± SEM. Unless otherwise noted, GraphPad Prism 5.0 was used for statistical analysis using One-Way ANOVA with Dunnett’s multiple comparison test and the means were considered significantly different when p ≤ 0.05.

## Results

### FFAR1 agonist TAK875 attenuates histamine-induced cortical tension development in HASM cells

**Fig. 1:**
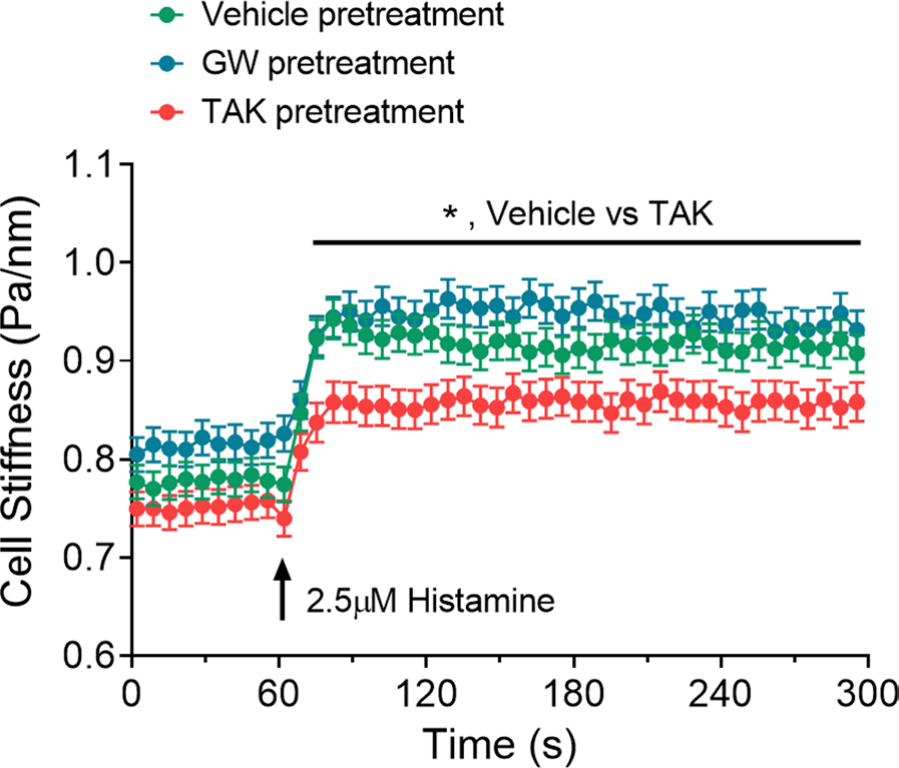
FFAR1 agonist reduced histamine-induced cell stiffness in HASM cells. HASM cells were derived from 2 lung donors. To control for random effects due to multiple cell measurements from the same donor, we applied mixed effect model using SAS V.9.2 (SAS Institute Inc., Cary, NC). HASM cells were pre-treated with vehicle (DMSO), GW9508 (10 µM), or TAK875 (10 µM) for 30 min. Following 30 min pretreatments, cell stiffness was measured for 60 s, and after histamine (2.5 µM) addition (t = 60 s), stiffness was continuously measured for the next 240 s. Data are presented as Estimated Mean ± SE, n = 267–278 cell measurements per treatments from 2 donors; p < 0.05: *)

To determine whether FFAR1 activation modulates cell shortening, we measured histamine-induced changes in the stiffness of HASM cells pre-treated with vehicle, GW9508 or TAK875 using MTC. A 30-min pretreatment with FFAR1 agonists had little effect on baseline cell stiffness (Fig. 1). Compared to vehicle treated cells, however, TAK875-treated cells showed a significant attenuation in histamine-induced increases in the cell stiffness (Fig. 1). GW9508 had little effect on histamine-induced cell stiffening responses (Fig. 1).

### FFAR1 agonist TAK875 attenuates histamine-induced MLC phosphorylation in HASM cells

**Fig. 2:**
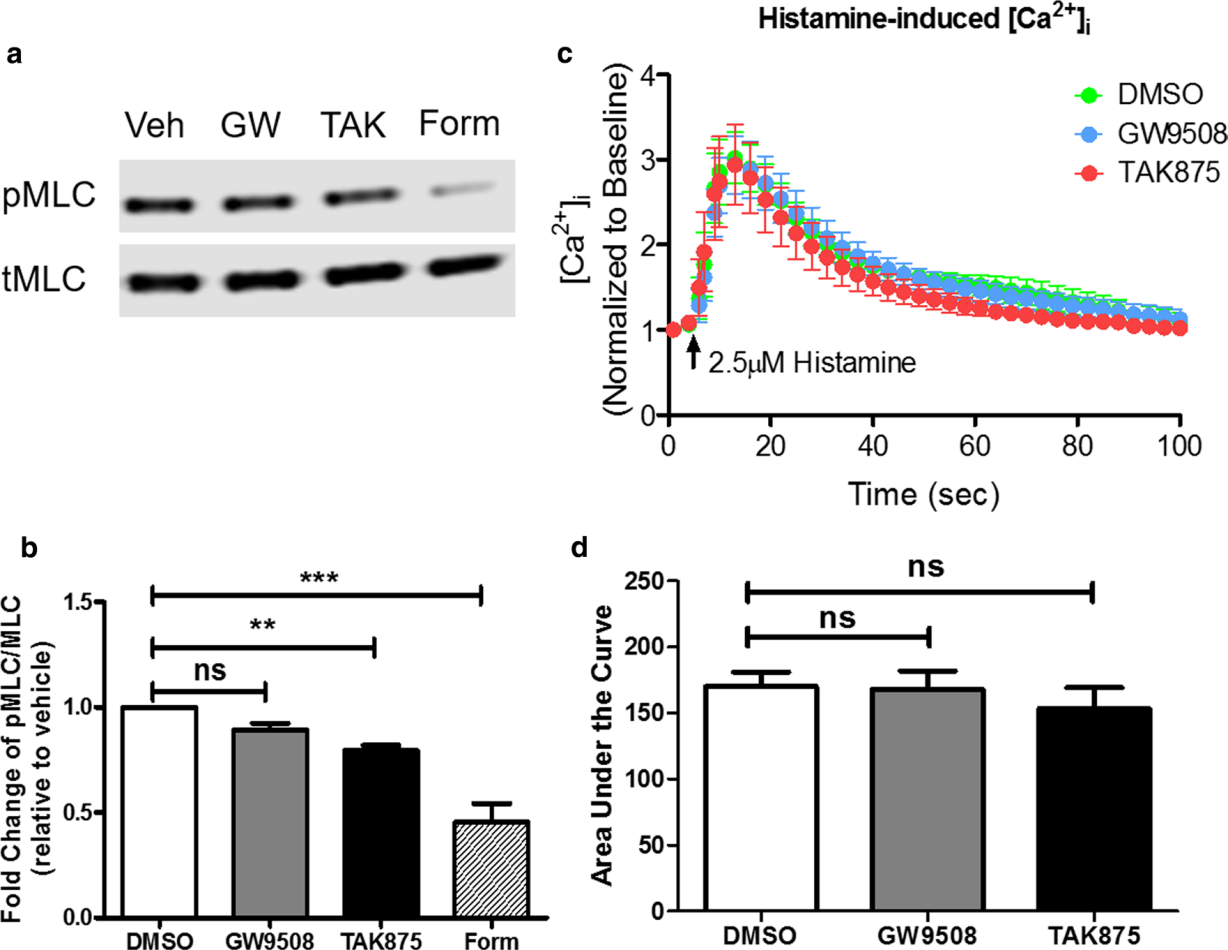
FFAR1 agonist TAK875 attenuates histamine-induced MLC phosphorylation in HASM cells. HASM cells were pre-treated with vehicle (DMSO), GW9508 (0.1–10 µM), TAK875 (10 µM), or Formoterol (10 nM) for 10–30 min, then stimulated with histamine 2.5 µM for 10 min. Agonists-induced MLC phosphorylation were determined. **a**,** b** 10 µM TAK875 pre-treatment for 30 min decreased histamine-induced MLC phosphorylation. (n = 7 donors) **c**, **d** 10 µM GW9508 or TAK875 pre-treatment has little effect on histamine-induced [Ca^2+^]_i_. (n = 6 donors) (One-way ANOVA with Dunnett’s test, compared to DMSO; *ns* not significant; p < 0.01: **, p < 0.001: ***)

Phosphorylation of MLC is the terminal event leading to ASM cell shortening. To determine whether attenuated MLC phosphorylation leads to reduced cortical tension development, we measured phosphorylated and total MLC levels in cells pre-treated with FFAR1 agonists. Consistent with MTC studies, TAK875, but not GW9508, significantly attenuated histamine-induced MLC phosphorylation (Fig. 2a, b). As a positive control, histamine-induced MLC phosphorylation was markedly inhibited by formoterol, a β_2_AR-acting bronchodilator. Together these results suggest that the bronchoprotective effect of TAK875 is mediated by inhibition of MLCK.

Agonist-induced rise in cytosolic Ca^2+^ activates MLCK to increase MLC phosphorylation. Interestingly, FFAR1 agonists had little effect on histamine-induced Ca^2+^ mobilization (Fig. 2c, d). These results suggest that the mechanistic effects of TAK875 on histamine-induced MLC phosphorylation is downstream of receptor coupling and Ca^2+^ signaling.

### FFAR1 agonists attenuate carbachol-induced MLC phosphorylation in HASM cells

**Fig. 3:**
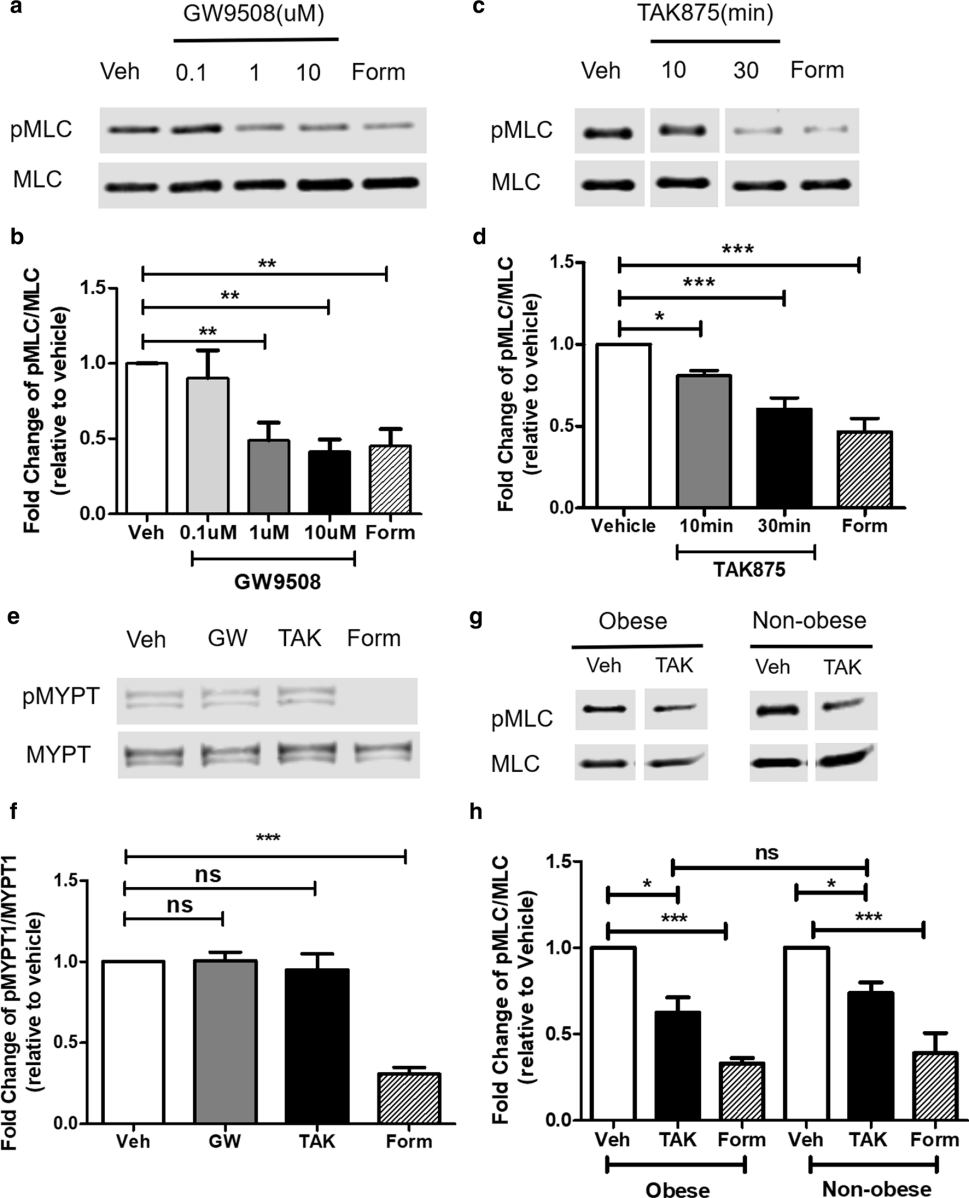
FFAR1 agonists attenuate carbachol-induced MLC phosphorylation in HASM cells. HASM cells were pre-treated with vehicle (DMSO), GW9508 (0.1–10 µM), TAK875 (10 µM), or Formoterol (10 nM) for 10–30 min, then stimulated with carbachol for 10 min. Agonists-induced MLC phosphorylation were determined. **a**, **b** 10 µM GW9508 pre-treatment for 30 min decreased CCh-induced MLC phosphorylation. (n = 5 donors) **c**, **d** 10 µM TAK875 pre-treatment for decreased CCh-induced MLC phosphorylation. (n = 7 donors) **e**, **f** 10 µM GW9508 and TAK875 pre-treatment for 30 min has little effect on CCh-induced MYPT1 phosphorylation. **g**, **h** TAK875 attenuated CCh-induced MLC phosphorylation to a comparable magnitude in non-obese and obese donor derived HASM cells (n = 3–5 donors). (One-way ANOVA with Dunnett’s Test, compared to Veh; *ns* not significant; p < 0.05: *, p < 0.01: **, p < 0.001: ***)

To determine whether the FFAR1 agonists’ effect is selective to contractile agonist receptor, we also measured carbachol (CCh)-induced MLC phosphorylation in HASM cells pre-treated with GW9508, TAK875 or vehicle. GW9508 and TAK875 significantly attenuated CCh-induced MLC phosphorylation (Fig. 3a–d). The decreases in CCh-induced MLC phosphorylation were comparable to that of cells pre-treated with formoterol, albeit with lower potency. The lower potency of FFAR1 agonists may be due to lower levels of FFAR1 mRNA expression in the majority of HASM cells tested (Additional file 2: Figure S1).

In HASM cells, muscarinic receptor activation increases myosin phosphatase target subunit 1 (MYPT1) phosphorylation to elicit ASM cell shortening via the activation of PI3K δ isoform, and phosphorylation of AKT [22]. GW9508 or TAK875 had little effect on CCh-induced MYPT1 phosphorylation, suggesting MLCP activity or RhoA-ROCK-mediated Ca^2+^ sensitization is not altered by FFAR1 agonists (Fig. 3e, f). Further, FFAR1 agonists had little effect on CCh-induced AKT phosphorylation, suggesting that the inhibitory effects of these FFAR1 agonists are independent of the PI3K/AKT activation (Additional file 3: Figure S2). We also investigated whether CCh-induced intracellular Ca^2+^ ([Ca^2+^]_i_) is altered by the FFAR1 agonists and found that they had little effect on this pathway (Additional file 4: Figure S3). In addition, TAK875 attenuated CCh-induced MLC phosphorylation to a comparable magnitude in non-obese and obese donor derived HASM cells (Fig. 3g, h).

### FFAR1 agonists manifest a broncho-protective effect in β_2_AR-desensitized HASM cells

**Fig. 4:**
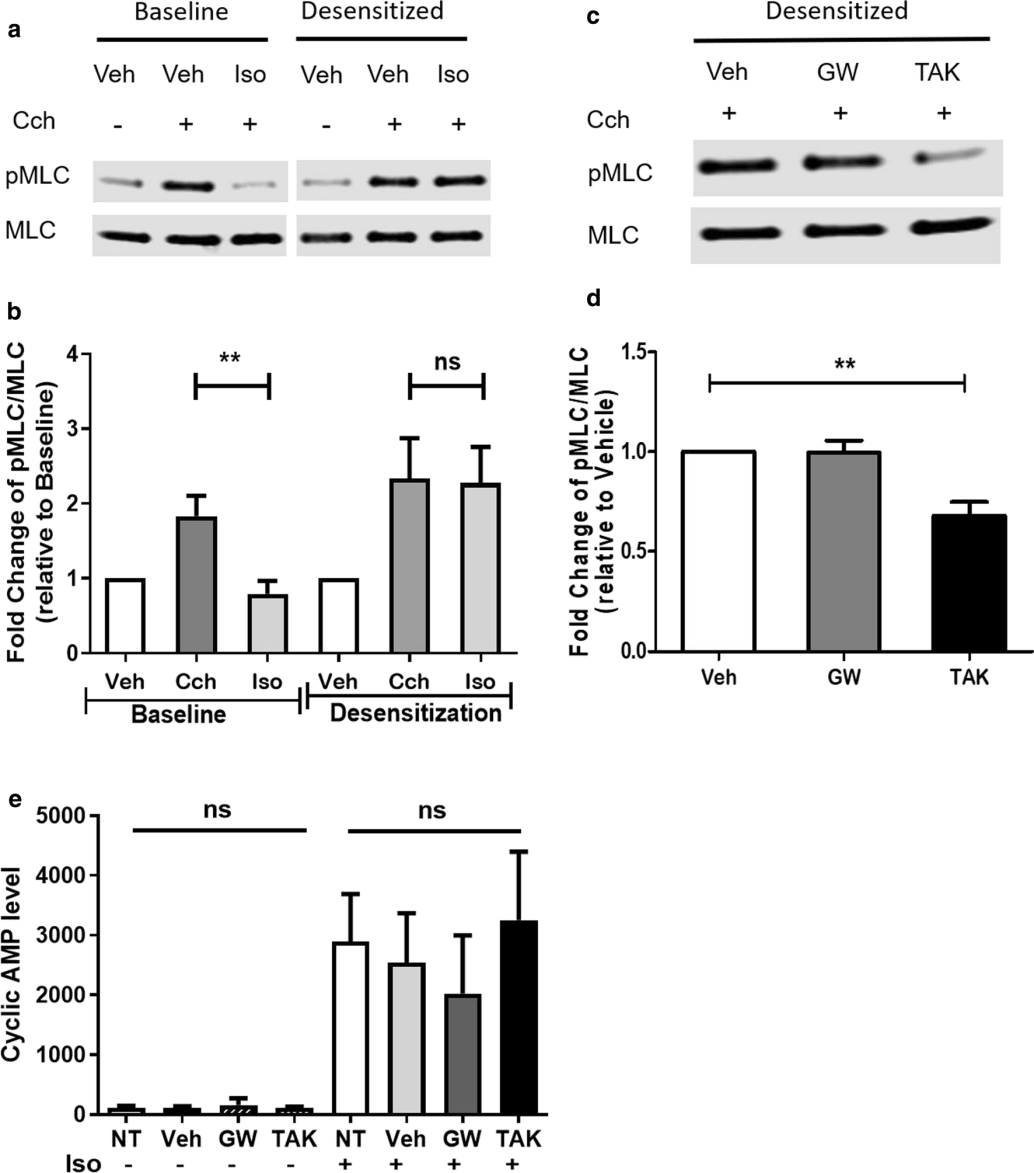
FFAR1 agonists manifest a broncho-protective effect in β_2_AR-desensitized HASM cells. HASM cells were desensitized by salmeterol (1 µM) for 18 h. Then, HASM cells pre-treated with vehicle (DMSO), GW9508 (10 µM), or TAK875 (10 µM) for 30 min, then stimulated with carbachol 25 µM for 10 min. Agonists-induced MLC phosphorylation were determined. **a**, **b** Isoproterenol decreased CCh-induced MLC phosphorylation, but it loses its bronchoprotective effects after salmeterol-induced β_2_AR desensitization. (n = 6 donors) **c**, **d** FFAR1 agonist TAK875 attenuated CCh-induced MLC phosphorylation in β_2_AR desensitized HASM cells. (n = 6 donors) **e** FFAR1 agonists have little effect on cyclic AMP production in HASM cells. HASM cells pre-treated with vehicle (DMSO), GW9508 (10 µM), or TAK875 (10 µM) for 30 min, then stimulated with or without isoproterenol for 5 min to induce cAMP production. Baseline and iso-induced cAMP levels were determined (n = 6 donors). (One-way ANOVA with Dunnett’s Test, compared to Veh; p < 0.01: **; *ns* not significant, *NT *no treatment)

Prolonged exposure to beta-2 adrenergic receptor (β_2_AR) agonists desensitizes the receptor and promotes tachyphylaxis [23]. To test whether these FFAR1 agonists retain broncho-protective effect in the presence of β_2_AR desensitization, HASM cells were treated with salmeterol (1 μM) for 18 h to induce β_2_AR desensitization, followed by exposure to CCh in the presence of vehicle, GW9508 or TAK875. FFAR1 agonist TAK875 attenuated CCh-induced MLC phosphorylation in both non-desensitized and desensitized HASM cells (Fig. 4c, d), while isoproterenol (10 μM) failed to attenuate CCh-induced MLC phosphorylation in desensitized HASM cells (Fig. 4a, b).

β_2_AR elicit ASM cell relaxation through increased intracellular cyclic AMP (cAMP). In light of our findings that FFAR1 agonists broncho-protect against β_2_AR desensitization, we tested whether these FFAR1 agonists modulate intracellular cAMP levels. In HASM cells, isoproterenol significantly increased cAMP levels, with little effect by GW9508 or TAK875 (Fig. 4e).

## Discussion

Elevated serum free fatty acid levels are associated with increased adiposity, insulin resistance and cardiovascular disease risk [24]. Evidence suggests that agonists of free fatty acid receptors FFAR1 and FFAR4 increased cytosolic Ca^2+^ and amplified ASM shortening [15]. These observations prompted us to explore whether FFAR1 amplifies E–C coupling in obesity [6]. Surprisingly, our results show that FFAR1 agonists GW9508 and TAK975 attenuated agonist-induced MLC phosphorylation while TAK875 decreased agonist-induced cell stiffness in HASM cells. These FFAR1 agonists, however, had little effect on cytosolic Ca^2+^ or MYPT1 phosphorylation.

The concentrations of TAK875 and GW9508 used in this study were based our preliminary experiments and those of others [15, 25]. TAK875 was developed as an anti-diabetic drug (Fasiglifam, Takeda Pharmaceutical Company Ltd., Kanagawa, Japan) and underwent extensive pharmacokinetic profiling. In healthy human subjects, maximal plasma concentrations (C_max_) of TAK875 reached 41.8 μM and 76.7 μM, following single daily oral dosing with 400 mg and 800 mg fasiglifam, respectively [26]. Therefore, the TAK875 concentration of 10 μM in our experiments appears physiologically relevant and pharmacologically achievable.

Intracellular elevation of Ca^2+^ is a key step in E–C coupling in HASM cells. Some allergens and inflammatory cytokines promote AHR by enhancing Ca^2+^ mobilization in ASM cells [27–29]. Therefore, we tested the hypothesis that FFAR1 agonists attenuate E–C coupling and cell stiffness by abrogating cytosolic Ca^2+^ levels. Since the broncho-protective effects of FFAR1 was independent of Ca^2+^ flux, we focused on two other pathways that could be modulated by FFAR1 receptors. We previously reported that muscarinic cholinergic receptor activation in HASM cells elicit cell shortening through G_α12_-coupled PI3K activation and Ca^2+^ sensitization [22]. This mechanism is unlikely to be the target of FFAR1 agonists since pAKT and pMYPT1 levels are unaltered by FFAR1 agonists. Others reported that activation of FFAR1 increases actin polymerization in ASM cells [15]. It remains to be seen whether these FFAR1 agonists, particularly TAK875, modulate actin polymerization in HASM cells to elicit bronchoprotective effects. Evidence suggests that FFAR1 activation enhanced HASM cell proliferation in a MEK/ERK- and PI3K/Akt-dependent manner [25]. While not measuring HASM cell proliferation, we showed unaltered Akt phosphorylation by FFAR1 agonists that suggests these agents are not mitogenic. We and others have shown that PI3K activation is necessary for HASM cell growth [22]. Generally, pro-contractile signaling overlaps and shares signaling entities with proliferative signaling in many cell types. Theoretically, the bronchoprotective effects of FFAR1 agonists should not induce HASM cell proliferation. Furthermore, TAK875 and GW9508 acting through FFAR1, elicited potent anti-proliferative effects in multiple types of human melanoma cells lines, suggesting that FFAR1 may play a complex role in proliferation that is cell and tissue specific [30].

Bronchodilators and corticosteroids are the critical components in mainstream asthma therapy. About 5–10% of asthma patients, with severe asthma, have suboptimal response to corticosteroids [31]. Further, β_2_AR receptor desensitization and tachyphylaxis leads to uncontrolled asthma symptoms [32]. Therefore, there is an unmet need for novel bronchodilators to expand the current repertoire of treatments available for asthma. The FFAR1 agonist TAK875 attenuated MLC phosphorylation in β_2_AR-desensitized HASM cells, indicating the potential of this compound to curb AHR in severe asthma.

ASM relaxation is mediated by a variety of cell signaling pathways activated by cAMP mobilizing agents. β_2_AR, the major receptor responsible for ASM relaxation, activates adenylate cyclase activity and cAMP levels to mediate PKA-dependent inhibition of MLC kinase. Other mechanisms, such as inhibition of phospholipase activity, Ca^2+^ mobilization and activation of large conductance K^+^ channels are also implicated in ASM relaxation [33]. Studies in pancreatic beta cells showed that, GW9508 activates ATP-sensing K^+^ channels (K_ATP_ channels) to inhibit membrane depolarization and insulin secretion [34]. In HASM cells, activation of K_ATP_ channels caused relaxation, suggesting that these channels are functionally similar to that of pancreatic beta cells [35]. The regulatory roles of FFAR1 agonists on membrane potential remain to be elucidated, as are the K_ATP_ channels and other previously unrecognized Ca^2+^-evoked HASM relaxation mechanisms.

Although this study originated from our interest in obesity, the FFAR1 agonist TAK875 has no differential effect on MLC phosphorylation in obese- and non-obese donor derived HASM cells. However, our in vitro findings may not predict whether FFAR1 function in HASM cells is altered in obesity in vivo. Plasma free fatty acids are elevated in obesity, therefore prolonged exposure to free fatty acids may desensitize and modulate FFAR1 functions in obese individuals [36, 37]. Studies using ectopic expression systems demonstrated ligand-dependent and -independent FFAR1 receptor internalization, suggesting a desensitization mechanism similar to that of β_2_AR tachyphylaxis [38]. Collectively, how changes associated with an obesity phenotype, such as inflammation, proliferation and cell metabolism, are differentially modulated by FFAR1 or FFAR4 in obesity remains unknown [39].

Our key findings identify a bronchoprotective role for these FFAR1 agonists. However, we acknowledge the following deficiencies in the study: (i) The expression levels of FFAR1 mRNA is low in HASM cells (Additional file 2: Figure S1). This low expression precluded siRNA-mediated silencing of FFAR1 as an additional approach to determine the necessity of this receptor in attenuated E–C coupling. It is plausible that the FFAR1 agonists elicit their bronchoprotective effect, not through the FFAR1 but through hitherto unidentified targets. (ii) We have not focused on the FFAR4 in this study [40]. Based on Ca^2+^ mobilization assays, GW9508 showed ~ 100-fold affinity to FFAR1 than FFAR4. The selectivity of GW9508 to FFAR1 was demonstrated in other cell types mostly in ectopic expression backgrounds [41]. It is plausible that the relative selectivity of GW9508 towards FFAR1 is different in endogenous expression conditions seen in HASM cells. In light of the recent report on FFAR4 and ASM relaxation, this needs to be addressed in future studies [18]. Although these limitations prevent us from confirming the necessity of FFAR1 to bronchoprotection, our findings suggest that TAK875 acts to reverse or prevent HASM shortening.

## Conclusion

**Fig. 5:**
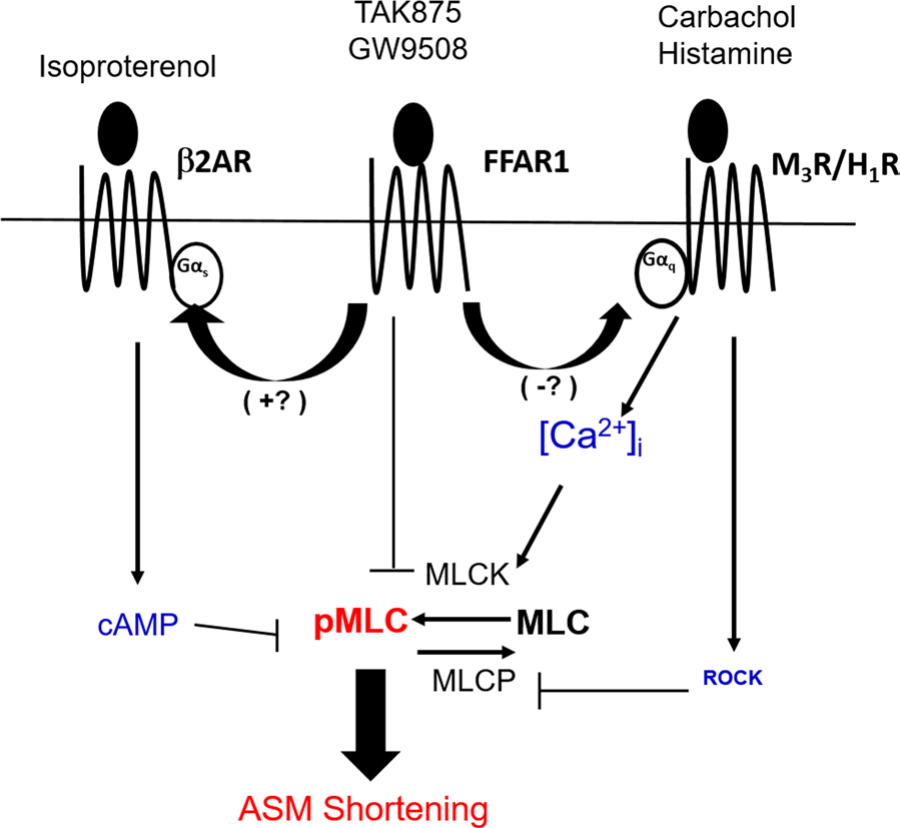
Conceptual model of FFAR1 agonist effect on some steps of EC coupling and cell shortening in HASM cells. The activation of FFAR1 receptors by agonists, GW9508 or TAK875, leads to the attenuation of MLC phosphorylation and cell shortening in HASM cells. The precise molecular mechanisms remain unknown but are independent of cAMP generation, pAKT inactivation, and MYPT1 phosphorylation. (blue font: no change by FFAR1 agonists; red font: attenuated by FFAR1 agonists)

FFAR1 agonists, GW9508 and TAK875, attenuate agonist-induced MLC phosphorylation while TAK875 decreases agonist-induced ASM cell shortening. The precise molecular mechanisms remain unknown but the effects are independent of cAMP generation, pAKT inactivation or MYPT1 phosphorylation (summarized in Fig. 5). There were no differential effects by FFAR1 agonist TAK875 in HASM cells derived from obese and non-obese donors. Arguably, FFAR1 activation can serve as a novel therapeutic target to broncho-protect human airways in airway diseases such as asthma and COPD.

## Supplementary information

**Additional file 1: Table S1**. Characteristics of human lung donors in the study. **Table S2. **Concomitant medications and morbidities of lung donors in the study.

**Additional file 2: Figure S1. **FFAR1 mRNA Expression in HASM cells. SYBR green-based qRT-PCR was performed to determine the expression level of FFAR1 (GPR40) in HASM cells. (A-B) C_T_ values of GPR40 and the house-keeping control cyclophilin in HASM cells.

**Additional file 3: Figure S2. **FFAR1 agonist effect on MLC phosphorylation is independent of AKT phosphorylation. HASM cells were pre-treated with vehicle (DMSO), GW9508 (0.1–10 μM), TAK875 (10 μM), or Formoterol (10 nM) for 10–30 min, then stimulated with carbachol 25 μM for 10 min. Agonist-induced Akt phosphorylation was determined. (A-B) 0.1–10 μM GW9508 pre-treatment for 30 min has little effect on CCh-induced AKT phosphorylation. (n = 5 donors) (C-D) TAK875 pre-treatment for 10 or 30 min has little effect on Cch-induced AKT phosphorylation. (n = 7 donors) (One-way ANOVA with Dunnett’s Test, compared to DMSO; p < 0.05: *).

**Additional file 4: Figure S3. **FFAR1 agonists have little effect on intracellular [Ca^2+^]_i_ in HASM cells. HASM cells were pre-treated with vehicle (DMSO), GW9508 (10 μM), or TAK875 (10 μM) for 30 min, then stimulated with carbachol 25 μM or histamine 2.5 μM. Agonists-induced [Ca^2+^]_i_ were determined for 100 s. (A-C) GW9508 or TAK875 pre-treatment has little effect on CCh-induced calcium mobilization. (n = 6 donors; One-way ANOVA with Dunnett’s Test, compared to DMSO; ns—not significant).

## Data Availability

The data generated and used during the current study are available from the corresponding author on reasonable request.

## Abbreviations

FFAR1: Free fatty acid receptor 1
FFAR4: Free fatty acid receptor 4
GPCR: G protein-coupled receptors
HASM: Human airway smooth muscle
AHR: Airway hyperresponsiveness
MLC: Myosin light chain
pMLC: Phosphorylated MLC
AKT: Protein kinase B
MLCK: Myosin light chain kinase
MLCP: Myosin light chain phosphatase
ROCK: Rho-associated kinase
E–C: Excitation–contraction
CCh: Carbachol
[Ca^2^^+^]_i_: Cytosolic Ca^2^^+^
MTC: Magnetic twisting cytometry
AUC: Area under the curve
cAMP: Cyclic adenosine monophosphate
MYPT1: Myosin phosphatase target subunit 1
pMYPT1: Phosphorylated MYPT1
M_3_: Muscarinic receptors subtype 3
H_1_: Histamine receptor subtype 1
β_2_AR: β2 Adrenergic receptor
K_ATP_: ATP-sensing K^+^ channels
