# Supplementary material for: FFAR1 activation attenuates histamine-induced myosin light chain phosphorylation and cortical tension development in human airway smooth muscle cells
Source: Respir Res. 2020 Nov 30;21:317. doi: 10.1186/s12931-020-01584-w (PMC7708129; doi:10.1186/s12931-020-01584-w)
Supplement: Supplementary file 1 — Additional file 1: Table S1. Characteristics of human lung donors in the study. Table S2. Concomitant medications and morbidities of lung donors in the study. [file 12931_2020_1584_MOESM1_ESM.docx]

**Table S1. Characteristics of human lung donors in the study**

| **Characteristics** | **Non-obese** | **Obese** | ***P* Value** |
| --- | --- | --- | --- |
| Sex, Female/Male | 5/0 | 5/0 | 1 |
| Mean Age, Yrs. (SEM) | 30.6 (7.1) | 36.0 (6.5) | 0.59 |
| Race, Caucasian/Black | 3/2 | 3/2 | 1 |
| Mean BMI, Kg/m^2^ (SEM) | 22.49 (0.86) | 43.32 (4.15) | 0.0012 |

**Table S2. Concomitant medications and morbidities of lung donors in the study.**

| **Donor#** | **Obese (O) / Non-Obese (N)** | **Cause of Death** | **Medications** | **Other Chronic Conditions** |
| --- | --- | --- | --- | --- |
| D1 | N | CVA | Metformin, Plavix, Lasix, Spironolactone | Type 2 Diabetes |
| D2 | N | CVA/Stroke | Xanax, Amitriptyline, ASA, Chlorthalidone, Colace, Neurontin, Hydralazine, Imdur, Labetalol, Lisinopril, Oxycodone, Simvastatin | Type 2 Diabetes |
| D3 | N | Head Trauma | None | Type 2 Diabetes |
| D4 | N | Head Trauma | None | None |
| D5 | N | Head Trauma | Prenatal Vitamins, Birth Control | None |
| D6 | O | Anoxia | Percocet, Tramadol | Depression |
| D7 | O | Head Trauma | Melatonin, Ferrous Sulfate | None |
| D8 | O | CNS Tumor | Steroids | None |
| D9 | O | CVA/Stroke | Dialysis | Migraine, Anemia, Hypertension, Renal Failure, Kidney Transplant Rejection, GERD |
| D10 | O | ICH/Stroke | Birth Control Implant, possibly Zoloft | Anxiety/Depression |

(CVA, cardiovascular accident; CNS, central nervous system; ICH, intra-cranial hemorrhage; GERD, gastroesophageal reflux disease).
