# Supplementary figures and images for: FFAR1 activation attenuates histamine-induced myosin light chain phosphorylation and cortical tension development in human airway smooth muscle cells
Source: Respir Res. 2020 Nov 30;21:317. doi: 10.1186/s12931-020-01584-w (PMC7708129; doi:10.1186/s12931-020-01584-w)

**A**

|        | C <sub>T</sub> Values |             |
|--------|-----------------------|-------------|
| Donors | GPR40                 | Cyclophilin |
| D1     | 34.025                | 24.435      |
| D2     | 32.25                 | 22.815      |
| D3     | 34.21                 | 23.04       |
| D4     | 31.265                | 18.88       |
| D5     | 29.6                  | 18.35       |

**B**

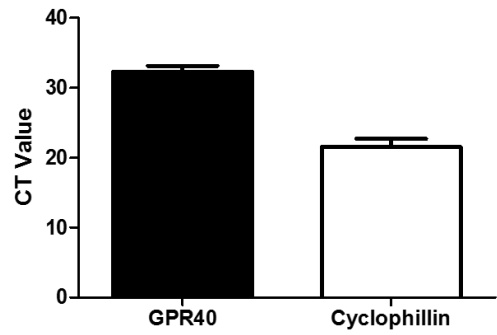

Supplement: Supplementary file 2 — Additional file 2: Figure S1. FFAR1 mRNA Expression in HASM cells. SYBR green-based qRT-PCR was performed to determine the expression level of FFAR1 (GPR40) in HASM cells. (A-B) CT values of GPR40 and the house-keeping control cyclophilin in HASM cells. [file 12931_2020_1584_MOESM2_ESM.pdf]

**A**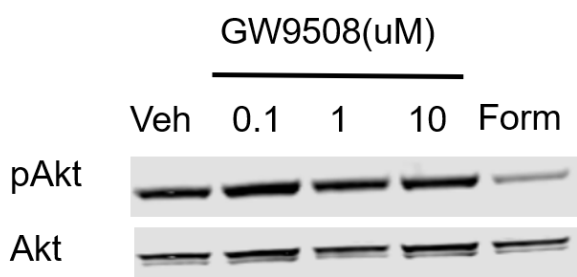**B**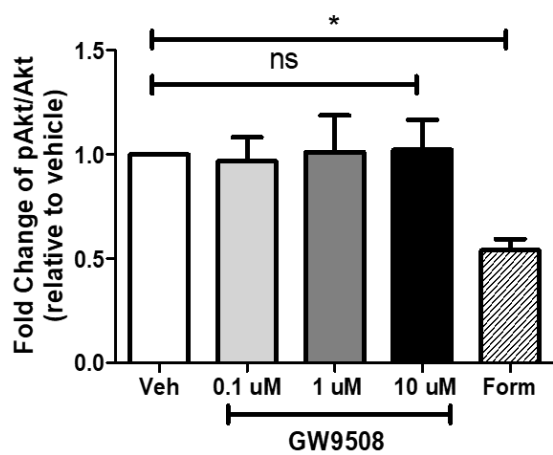**C**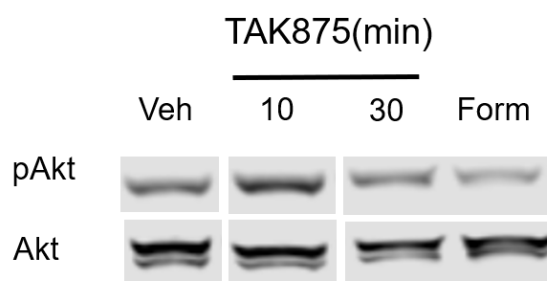**D**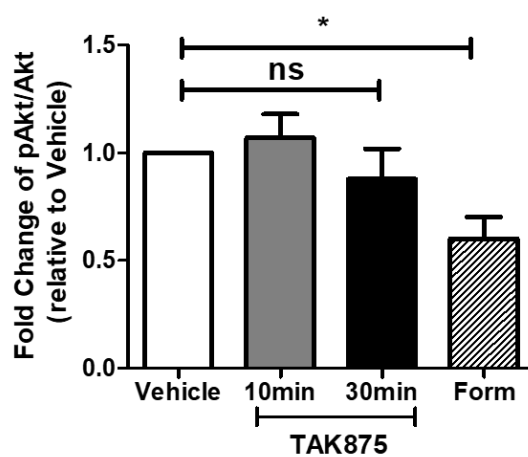

Supplement: Supplementary file 3 — Additional file 3: Figure S2. FFAR1 agonist effect on MLC phosphorylation is independent of AKT phosphorylation. HASM cells were pre-treated with vehicle (DMSO), GW9508 (0.1–10 μM), TAK875 (10 μM), or Formoterol (10 nM) for 10–30 min, then stimulated with carbachol 25 μM for 10 min. Agonist-induced Akt phosphorylation was determined. (A-B) 0.1–10 μM GW9508 pre-treatment for 30 min has little effect on CCh-induced AKT phosphorylation. (n = 5 donors) (C-D) TAK875 pre-treatment for 10 or 30 min has little effect on Cch-induced AKT phosphorylation. (n = 7 donors) (One-way ANOVA with Dunnett’s Test, compared to DMSO; p < 0.05: *). [file 12931_2020_1584_MOESM3_ESM.pdf]

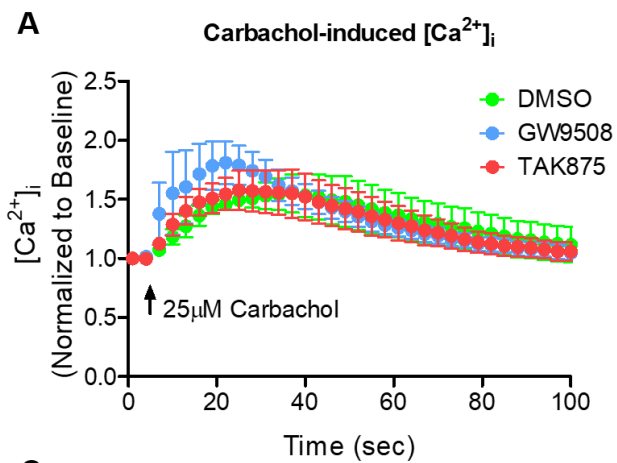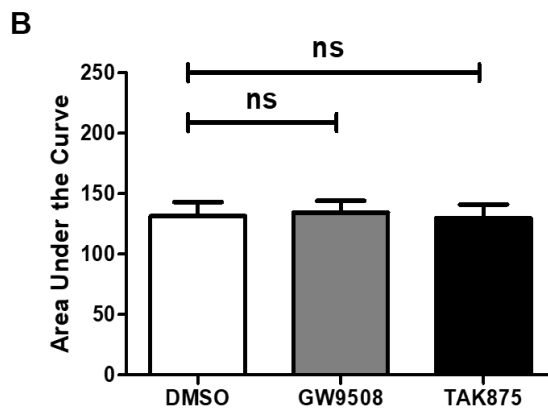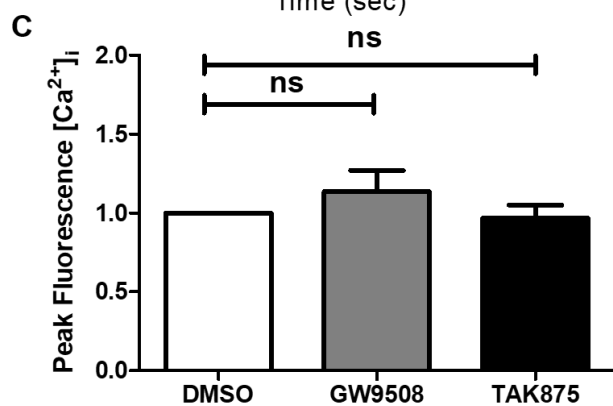

Supplement: Supplementary file 4 — Additional file 4: Figure S3. FFAR1 agonists have little effect on intracellular [Ca2+]i in HASM cells. HASM cells were pre-treated with vehicle (DMSO), GW9508 (10 μM), or TAK875 (10 μM) for 30 min, then stimulated with carbachol 25 μM or histamine 2.5 μM. Agonists-induced [Ca2+]i were determined for 100 s. (A-C) GW9508 or TAK875 pre-treatment has little effect on CCh-induced calcium mobilization. (n = 6 donors; One-way ANOVA with Dunnett’s Test, compared to DMSO; ns—not significant). [file 12931_2020_1584_MOESM4_ESM.pdf]
